# Measuring Mind Wandering During Online Lectures Assessed With EEG

**DOI:** 10.3389/fnhum.2021.697532

**Published:** 2021-08-09

**Authors:** Colin Conrad, Aaron Newman

**Affiliations:** ^1^School of Information Management, Faculty of Management, Dalhousie University, Halifax, NS, Canada; ^2^Department of Psychology and Neuroscience, Faculty of Science, Dalhousie University, Halifax, NS, Canada

**Keywords:** electroencephalography, mind wandering, e-learning, online lecture, asynchronous lecture, remote learning

## Abstract

Mind wandering can inhibit learning in multimedia classrooms, such as when watching online lectures. One explanation for this effect is that periods of mind wandering cause learners’ attention to be redirected from the learning material toward task-unrelated thoughts. The present study explored the relationship between mind wandering and online education using electroencephalography (EEG). Participants were asked to attend to a 75 minute educational video lecture, while task-irrelevant auditory tones played at random intervals. The tones were of two distinct pitches, with one occurring frequently (80%) and the other infrequently (20%). Participants were prompted at pseudo-random intervals during the lecture to report their degree of experienced mind wandering. EEG spectral power and event-related potentials (ERP) were compared between states of high and low degrees of self-reported mind wandering. Participants also performed pre/post quizzes based on the lecture material. Results revealed significantly higher delta, theta and alpha band activity during mind wandering, as well as a decreased P2 ERP amplitude. Further, learning scores (improvement on quizzes pre to post) were lower among participants who reported higher degrees of mind wandering throughout the video. The results are consistent with a view that mind wandering during e-learning is characterized by a shift in attention away from the external world and toward internal thoughts, which may be a cause of reduced learning.

## Introduction

In 2020 higher learning institutions across the world quickly transitioned their teaching to an online format, in response to social distancing requirements enacted to limit the spread of COVID-19. Though in the early days of the outbreak many instructors adopted synchronous online lecture course formats, there were soon calls in the higher education community to adopt asynchronous activities, as awareness was raised about the limitations of synchronous online lectures (Flaherty, 2020). Many universities and colleges have since adopted pre-recorded, asynchronous lectures, which are often viewed as a more accessible alternative. However, there is evidence to support that online lectures, particularly when they are pre-recorded, do not benefit students similarly to their in-person equivalent (Williams et al., 2012) potentially because they do not facilitate student engagement to the same degree (O’Callaghan et al., 2017).

One of the ways that pre-recorded online lectures may fail to replicate in-person experiences is that students’ minds are more likely to wander (Szpunar et al., 2013). Mind wandering is a phenomenon characterized by a shift in attention away from a primary task, toward unrelated self-generated thoughts (Smallwood and Schooler, 2006, 2015). It has been found to impact performance on monotonous tasks, such as driving long distances (Baldwin et al., 2017; Zhang and Kumada, 2017) or learning from long texts or long lectures (Wammes and Smilek, 2017; Forrin et al., 2020). It follows that students who experience mind wandering learn less, as their attention is directed away from the material they are supposed to learn. Some scholars have concluded that teaching practices should therefore be developed to prevent mind wandering (Smallwood and Schooler, 2015). Online lectures might similarly benefit by incorporating design principles that limit mind wandering and/or provide corrective feedback when it occurs.

The mind wandering phenomenon can be incorporated into existing models of executive attention and control, and the neuroimaging techniques for measuring them (Smallwood and Schooler, 2006). Past research on the presence of mind wandering during meditation tasks have suggested that the regulation of attention is linked to heightened activity in the prefrontal cortex and anterior cingulate cortex (Hasenkamp et al., 2012; Xu et al., 2014). We can posit that there may be a similar link in the online lecture context and that it is measurable.

However, it is difficult to identify which pre-recorded or online lecture designs inhibit mind wandering because of the difficulty of measuring the mind wandering phenomenon in the first place. Although there is some evidence to suggest that *ex post* questionnaires (i.e., administered after a learning video) can effectively measure the amount of subjective mind wandering (Mrazek et al., 2013), other research suggests that mind wandering episodes are forgotten over time (Weinstein, 2018), and may exhibit variations throughout a lecture. Experience sampling is an alternative approach, in which people are prompted to respond to questions about their state of mind wandering at intervals throughout a task. However, this disrupts both mind wandering and the target task that the mind wandering occurs during (Schooler, 2004; Wammes and Smilek, 2017). It is desirable to identify an alternative approach which does not disrupt mind wandering or task performance, while also giving insights into the cognitive mechanisms behind the phenomenon. One potential approach is using electroencephalography (EEG), which monitors brain activity during an activity, in real time, without disrupting the activity as experience sampling does.

There is considerable emerging literature on potential EEG markers of mind wandering. Dhindsa et al. (2019) studied the EEG correlates of mind wandering during live lectures and found an association between mind wandering and decreased oscillatory activity at the alpha band in the occipital region, which they interpreted as an indicator of decreased attention. Other EEG studies have similarly found associations between mind wandering and attentional disengagement with stimulus processing. Braboszcz and Delorme (2011) identified two varieties of EEG measures which were associated with mind wandering during a meditation task. First, they investigated oscillatory effects, and noted increased frontal delta and theta, as well as decreased occipital alpha power during mind wandering. Second, they observed increased amplitude of the attention-related P2 event-related potential (ERP) component time-locked to auditory stimuli during reported states of mind wandering. These findings have been corroborated by further work which found theta power to be a reliable measure of mind wandering generally (van Son et al., 2019) as well as increased P2 amplitude (Xu et al., 2018).

However, other studies have found conflicting results. A series of studies have observed that task-related stimuli elicited decreased P1 amplitudes (Kam et al., 2011; Baird et al., 2014) as well as decreased P3 amplitudes (Kam et al., 2011, 2014; Baldwin et al., 2017) during states of mind wandering. Furthermore, subsequent studies which employed a random prompt experience sampling method during monotonous active attention tasks—rather than the button press method and meditation task described by Braboszcz and Delorme (2011)—observed increased, rather than decreased, alpha during reported mind wandering (Baird et al., 2014; Baldwin et al., 2017; Compton et al., 2019; Arnau et al., 2020). Collectively, these results suggest that the nature of a task or experience sampling method may affect how mind wandering affects ERP amplitudes.

In this study, we sought to identify EEG markers of mind wandering during an online lecture task which required sustained attention. We designed an experiment which administered frequent and infrequent auditory stimuli (an “oddball” paradigm) which participants were instructed to ignore (Squires et al., 1975; Braboszcz and Delorme, 2011). Participants also underwent experience sampling and were prompted to report their degree of mind wandering at pseudo-random intervals throughout the lecture (Wammes and Smilek, 2017). Following Braboszcz and Delorme (2011), we compared EEG responses to auditory tones in a period immediately preceding periods of heightened mind wandering, to those preceding on-task thought. Participants were also given quizzes on the lecture content both before and after the lecture, and an *ex post* self-report questionnaire. Based on the work of Sullivan et al. (2015) and the NASA Task Load Index (NASA TLX 1989), the questionnaire measures were administered to identify whether there was an effect of task load or whether the reported mind wandering was related to an information technology. This latter question was included in response to calls by information technology scholars to investigate whether intended or actual technology use affects the degree or quality of mind wandering (Oschinsky et al., 2019; Klesel et al., 2021).

Our study employed task-unrelated auditory stimuli most similarly to Braboszcz and Delorme (2011), who observed a heightened P2 response to both standard and oddball task-unrelated tones during periods of mind wandering. Unlike that study and more similarly to later studies such as Compton et al. (2019) and Arnau et al. (2020), we employed an experience sampling method. We thus hypothesized that increased P2 amplitude, as well as increased delta, theta and alpha activity would be markers of mind wandering in a sustained e-learning task. We also predicted that self-reported mind wandering would be negatively correlated with online lecture learning outcomes. Such results would provide evidence that mind wandering is related to changes in attention, that these changes have an impact on learning during online lectures. It would also suggest markers of mind wandering which could be used to evaluate online lecture design in the future.

## Methods

### Participants

Fifty-two students (36 women and 16 men, aged 17–28 years; *M* = 20.6, *SD* = 2.5) gave written consent to participate in the experiment. Five participants’ data from the EEG analyses are not reported here due to technical errors with the recording, leaving a sample size of 48 individuals. Participants were excluded from the study if they were not fluent in English, were taking medication that could lead to abnormal EEG, or identified as having neurological disorders. Participants were also excluded if they had taken a course in venture capital, the subject of the learning video. Participants provided written and informed consent and were compensated in course credit or CAD $25 for their time. All procedures were reviewed by the Dalhousie University research ethics board, according to the Canadian Tri-Council Policy Statement and the Declaration of Helsinki.

### Stimuli

The teaching video was a 75-min English language video about venture capital (Fu, 2017). The subject matter and video were chosen because it was on a subject not commonly taught to our subject population (who comprised mainly psychology and neuroscience students, and who were screened to have no knowledge of the topic). The video consisted exclusively of two lecturers talking, and questions from the lecture hall audience. Pilot testing suggested that this video would trigger variations in mind wandering and attention for most participants.

The auditory stimuli were tones of 100 ms duration; standard (frequently presented) tones were 500 Hz and oddball (infrequent) tones were 1,000 Hz.

The quiz to assess learning of the lecture content was developed by the research team, and consisted of 10 multiple-choice questions based on content from the video. The quiz was administered before and after the video. The *ex post* questionnaire consisted 25 items including degree of task load (NASA TLX, 1988), the degree of experienced mind wandering related to technology (Sullivan et al., 2015), and sources of experienced mind wandering unrelated to technology (Sullivan et al., 2015). Additional items to measure interest in the course material and perception of attention throughout the video were also added.

### Procedure

After providing informed consent, participants were fitted with the EEG cap and were brought to the testing room. Participants completed the pre-study quiz and then were instructed to pay attention to the video and ignore the audio tones. Once EEG recording commenced, the video was started, and tones were played such that they were distinguishable over the lecture audio track. Tones were presented at intervals chosen randomly from a uniform distribution (1.0–1.5 s; mean 1.25 s), the order of standard and oddball tones was randomized, constrained such that 80% of the tones were standards and 20% oddballs. Ten mind wandering prompts were presented at pre-determined intervals throughout the video, with the intervals between prompts being selected from a uniform random distribution ranging from 1 to 16 min. The timing of the prompts was the same across all participants; however, because the lecture video and prompt presentation were started independently, the prompts occurred at approximately, but not precisely, the same time in the video for each participant.

At each prompt, participants were asked to report their degree of mind wandering or on-task experience from the time period immediately before the mind wandering prompt (Wammes and Smilek, 2017). The options were structured in a 5-point Likert-like scale ranging from “completely on task” to “completely mind wandering.” Stimulus presentation was controlled by a personal computer running the Windows 8 operating system. The video was played using Windows Media Player, while presentation of auditory tones, and collection of manual responses, was controlled by code written in the PsychoPy library (version 1.81; Peirce, 2007). Videos were presented on a ViewSonic VS 16265 video monitor located 32 cm from the participant’s face. Audio was delivered through Mackie MR5 MKIII speakers connected through a Mackie ProFX8 mixing board, which performed digital-to-analog conversion of the audio. Following the study, the *ex post* questionnaire was administered, followed by the post-study quiz.

### EEG Recording

Participants were fitted with 32 scalp electrodes (ActiCap, BrainProducts GmbH, Munich, Germany) positioned at standard locations in a soft cap according to the International 10-10 system and referenced during recording to the average of all electrodes. Bipolar recordings were made between the outer canthi of the two eyes and above and below one eye, to monitor for eye movements and blinks. Electrode impedances were kept below 30 kOhm throughout the experiment. Electroencephalography data were sampled at 512 Hz using Refa8 amplifier (Advanced NeuroTechnologies, Enschende, Netherlands), bandpass filtered between 0.01 and 170 Hz, and saved digitally using the ASAlab software (Advanced NeuroTechnologies). The identity of each audio tone (standard/oddball) was communicated to the EEG amplifier via TTL codes sent from PsychoPy via the parallel port (Peirce, 2007). To precisely synchronize the onset timing of each auditory tone with the EEG system, a custom-built, Arduino-based device (Baker, 2013) was used which took its input from the audio output of the mixing board that also fed the speakers, and sent a TTL pulse to the EEG system every time a voltage deflection (sound onset) was detected.

### Artifact Correction and Data Processing

The MNE-Python library (Gramfort et al., 2013, 2014) was used for all EEG data preprocessing. The onset of each audio event was defined by the timing of the signals from the Arduino device, with the identity of the tone type (standard/oddball) defined by the event code sent immediately prior to sound onset. For ERP analysis, a 0.1–40 Hz bandpass filter was applied to the data, followed by manual identification and removal of electrodes and epochs with excessive noise. The data were then segmented into epochs spanning 200 ms prior to the onset of each auditory tone, to 1 s after. Independent components analysis was then used to identify and remove artifacts such as eye blinks and eye movements (Delorme and Makeig, 2004) using the FastICA algorithm (Hyvarinen, 1999). Following ICA artifact correction, data were re-referenced to the average of the two mastoid electrodes (TP9 and TP10). EEG data were analyzed for stimuli occurring from 0 to 20 s before a mind wandering prompt, and labeled based on user responses to the prompts (i.e., a 5-point Likert scale). For ERPs, epochs were analyzed in the time domain by calculating the average amplitude during the component time windows (see below). Oscillatory analyses were performed by transforming the time-locked epoch data into the frequency domain using Morlet wavelets with 50 log-spaced frequencies ranging from 2 to 30 Hz with 1 cycle at the lowest frequency increasing linearly to a maximum of 15 cycles at the highest frequency. We also used Welch’s (1967) method to compare mean power spectrum density (PSD) from a subset of the epoch representing the 1 s post auditory stimulus from the delta (2–4 Hz), theta (4–7 Hz), alpha (8–12 Hz), and beta (13–30 Hz) frequency bands.

### Statistical Analysis

Given that there were exactly 10 mind-wandering prompts for each participant, there was no variability in the number of responses, though there was variability in the degree of mind wandering reported. Data from 4 participants were excluded due to technical issues in their recording. This resulted in a total of 5525 epochs between the 10 conditions (2 tone types × 5 levels of mind wandering).

We predicted the effect of the P2 component and chose the time windows of 225–275 ms, based on a prior study with a similar paradigm (Conrad and Newman, 2019). After assessing the grand average waveforms from the present study, however, we realized that the timings from the prior study did not generalize—likely due to changes in stimulus presentation parameters between experiments. We thus selected new time intervals for statistical analysis, based on visual inspection of the present dataset. We also observed visual differences between conditions in the N1 component immediately preceding the P2, and so analyzed data in that time window as well, as a *post hoc* exploratory analysis. Dependent measures for ERP analysis were mean amplitudes over the 75–125 ms (for the N1) and 150–200 ms (for the P2) intervals, over a frontal region of interest (including electrodes Fz, F3, F4, FC3, FC4, Cz, C3, and C4).

For oscillatory analysis, the dependent measures were the power in each of the frequency bands of interest centered on two regions chosen on the basis of past EEG results cited in the Introduction: a frontal region (including electrodes Fz, Fp1, Fp2, F3, F4) and an occipital region (including electrodes POz, Oz, O1, and O2).

Analyses of self-report measures on learning performance were conducted using simple linear regression with the improvement in quiz scores as the dependent variable. All statistical analysis on EEG data was performed using linear mixed effects (LME) using the R language (version 3.6.1) the *mgcv* library (Wood, 2021). The model’s fixed effects included reported mental state (5-point scale) and stimulus type (standard, oddball). Akaike information criterion (AIC) was used to find random effects models that carried the most information; random effects included by-subject slopes for mental state and stimulus type, as well as random intercepts for each subject. Random effects of electrode location, though not stimulus by subject, were included in the PSD comparisons, and were interpreted for significance using the Bonferroni-Holm correction.

## Results

### Mind Wandering

We collected 480 responses to experience sample probes from the 48 participants whose data is included in the study of which 112 corresponded to “completely on task” (Level 1 on a Likert scale), 149 to “somewhat on task” (Level 2 on a Likert scale), 110 to “neither mind wandering nor on task” (Level 3 on a Likert scale), 81 to “somewhat mind wandering” (Level 4 on a Likert scale), and 28 to “completely mind wandering” (Level 5 on a Likert scale). In line with Wammes and Smilek (2017), we observed increased degrees of mind wandering as the lecture progressed, with a pronounced difference between samples collected at the 15- and 30-min marks and a significant linear relationship between degree of reported mind wandering and elapsed lecture time (*t* = 7.541; *p* < 0.001).

### Learning Measure

Participants’ scores on the quiz assessing their knowledge of the lecture content were significantly higher after watching the video (*M* = 4.82; *SE* = 2.18) than before (*M* = 2.86; *SE* = 1.27; *t* = 5.13, *p* < 0.001), which suggests that participants attended to, and learned from the video. However, in both the pre- and post-lecture quizzes, participants correctly answered fewer than 50% of the 10 questions asked. Linear regression analysis revealed a significant negative relationship between *ex post* technology-unrelated mind wandering measures and improvement of quiz scores [*F*(1, 46) = 4.361; *p* = 0.0423; *R*^2^ = 0.067], though neither technology-related mind wandering [*F*(1, 46) = 1.458; *p* = 0.2335l; *R*^2^ = 0.009], nor task load [*F*(1, 46) = 0.2776; *p* = 0.6008; *R*^2^ = −0.01561] were found to be a significant predictor of quiz score improvement.

### ERPs

**FIGURE 1:**
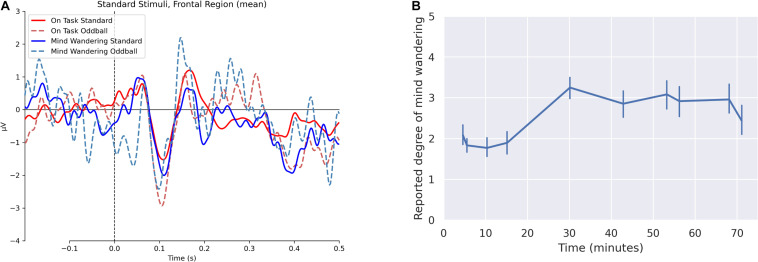
**(A)** Effect of the reported mind wandering states (i.e. “completely on task”, “completely mind wandering”) on event-related potentials elicited by standard and oddball stimuli. Images depicted are grand average waveforms at channels Fz, F3, F4, FC3, FC4, Cz, C3, and C4 for the two states. **(B)** Variances of self-reported mind wandering over time, with 95% confidence interval error bars.

The grand average ERP waveforms are illustrated in Figure 1 for the region of interest analyzed, which comprised electrodes over the anterior-central midline. We observed ERP components corresponding to the P1-N1-P2 complex, which varied in amplitude between conditions. These included a positive component peaking around 50 ms, a negative component peaking around 100 ms, and then a positive component peaking around 175 ms.

**FIGURE 2:**
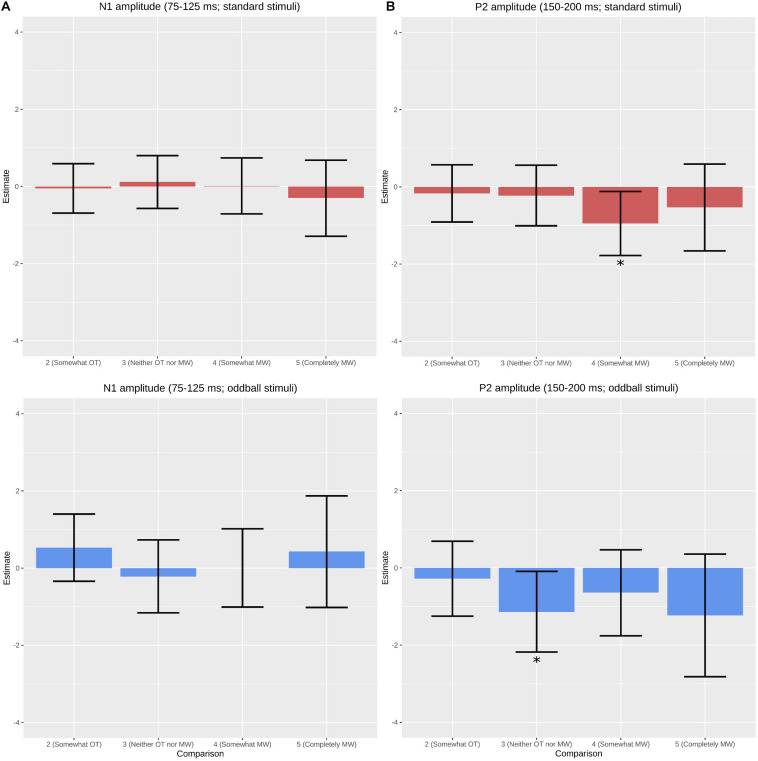
Comparisons of event-related potential estimates from linear mixed effects analysis using the “completely on task” state as the reference variable. **(A)** Responses to standard stimuli at the 75–125 ms window were not significantly different during the various reported mind wandering states, though responses to oddball stimuli were significantly lower. **(B)** Responses to standard stimuli at the 150–200 ms windows were consistently lower, though only significantly so during the “somewhat mind wandering” state. *Denotes significant at α = 0.05.

Results of the LME comparisons of ERPs are provided in Figure 2. For mind wandering, ERP amplitudes for each self-reported level of mind wandering were tested for differences against the reference condition of 1 (“completely on task”). In the 150–200 ms P2 time window (our *a priori* component of interest), analysis revealed a significantly more negative amplitude for standard stimuli during states reported at level 4 (“somewhat mind wandering”) relative to level 1 (β = −0.953; *t* = −2.88; *p* = 0.0041). We also observed significantly lower amplitude generated by oddball stimuli (β = −1.135; *t* = −2.71; *p* = 0.0067) during states reported at level 3 (“neither on task nor mind wandering”) relative to level 1. No other effects of mind wandering were significant.

### EEG Oscillatory Data

**FIGURE 3:**
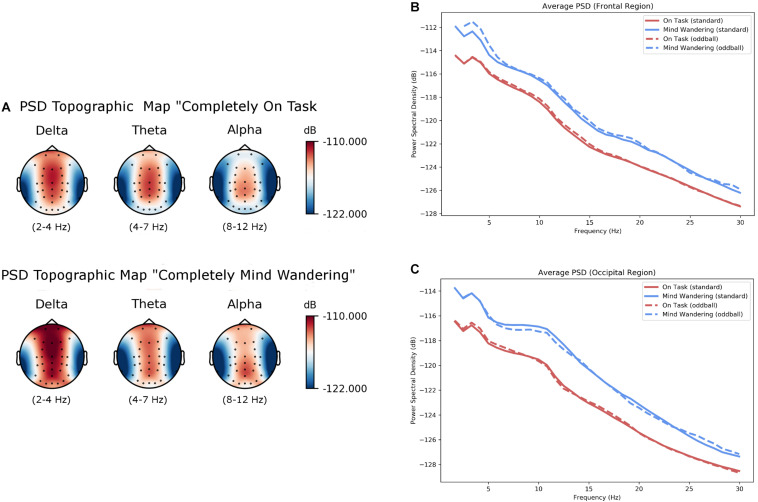
Effect of the extremes of the mind wandering states (“completely on task,” “completely mind wandering”) on power spectral density (PSD). **(A)** Topographic illustrations of PSD for the two states illustrate differences in delta and theta power in the frontal region, as well as increased alpha in the occipital region. **(B)** Average PSD in response to various stimuli are illustrated for channels Fz, Fp1, Fp2, F3, and F4 are represented. **(C)** Average PSD in response to various stimuli are again represented but for channels Poz, Oz, O1, and O2.

**FIGURE 4:**
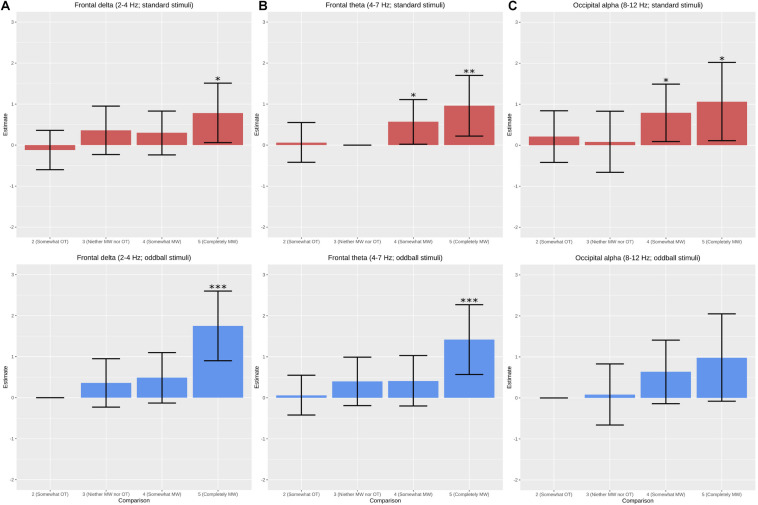
Comparisons of frequency band power (dB) estimates from linear mixed effects analysis using the “completely on task” state (1 on the Likert scale) as the reference variable. **(A)** Delta frequency band power in the frontal region is significantly higher during the “completely mind wandering” state and in response to oddball stimuli. **(B)** Frontal theta power is significantly higher during both “somewhat” and “completely” mind wandering states. **(C)** Alpha power in the occipital region is significantly higher during “somewhat” and “completely” mind wandering states though was only found to be significant in response to standard auditory stimuli. No significant results were found at the beta frequency band. *Denotes significant at α = 0.05; **denotes significant at α = 0.01; ***denotes significant at α = 0.001.

Power spectral density is represented as topographic maps and value by frequency in Figure 3. Results from LME analysis on oscillatory activity are summarized in Figure 4, which shows that power in all three frequency bands of interest (delta, theta, alpha) increased steadily as self-reported level of mind wandering increased. We did not observe any apparent effects in beta band power, nor were any statistically significant findings found for this frequency band, so we will not discuss it further. Analysis of band power over the 1 s windows revealed increased delta power in the frontal region during states reported at level 5 (“completely mind wandering”) relative to those reported at level 1 (“completely on task”; β = 0.938; *t* = 3.24; *p* = 0.001). We similarly observed significantly greater frontal theta band power during states reported at both level 4 (“somewhat mind wandering”; β = 0.543; *t* = 2.52; *p* = 0.011) and level 5 (β = 1.035; *t* = 3.52; *p* < 0.001) when compared to level 1. Significantly greater occipital alpha band power was observed during states reported at both level 4 (β = 0.763; *t* = 2.747; *p* = 0.002) and level 5 (β = 1.051; *t* = 2.77; *p* = 0.0055), when compared to level 1. Across all three frequency bands, the significant effects reflected increasing power with heightened degrees of mind wandering.

## Discussion

In this study we combined EEG recording with experience sampling, a pre/post learning quiz, and an *ex post* questionnaire to assess mind wandering while people watched an online lecture. During the lecture, task-irrelevant auditory tones were played, with two different pitches occurring with different probabilities (80 vs. 20%). The auditory tones served as attention probes to which ERPs were time-locked. Following previous literature, we predicted a larger P2 ERP component to both standard (80% frequency) and oddball (20% frequency) tones during mind wandering relative to when people were on task. We also predicted increased delta, theta, and alpha band power during mind wandering.

Our ERP results were not consistent with our hypotheses. During mind wandering, P2 amplitudes were actually lower than when on task, though not consistently across the various degrees of mind wandering. The facts that these effects were opposite to those predicted, occurred only at specific self-reported levels of mind wandering, that these were not the highest levels of mind wandering reported, and that the significant effects did not occur at the same levels of mind wandering across standard and oddball stimuli, all point to weak and possibly non-reproducible effects.

One possible reason for significant effects only at moderate levels of mind wandering (3 and 4 on our 5-point scale) and not at the “completely mind wandering” level is that there were far fewer trials at the highest level of mind wandering. If this is the case, the results are consistent with studies that observed decreased ERP amplitudes to task-related stimuli (Kam et al., 2011, 2014; Baird et al., 2014; Baldwin et al., 2017) and that the participants associated the tones with the e-learning task. It is thus possible that the pattern observed in our study similarly reflects a sort of “tuning out” of the outside world as attention drifts away from the task and toward unrelated thoughts. Further data will be required to determine whether the P2 is a reliable indicator of mind wandering in tasks requiring attention to external stimuli.

In contrast, we corroborated past frequency domain findings, namely that of increased frontal delta, theta and alpha band power, as observed by studies which employed experience sampling probes (Baird et al., 2014; Baldwin et al., 2017; Compton et al., 2019; Arnau et al., 2020). While some past studies such as those by Braboszcz and Delorme (2011) and van Son et al. (2019) observed decreased alpha while mind wandering, these studies employed a button press method that required participants to self-identify times that their mind was wandering, rather than random prompt sampling technique. Furthermore, these studies observed mind wandering during meditation tasks in which participants’ eyes are closed, in contrast with the later studies which required participants to have their eyes open and remain vigilant. Seli et al. (2015, 2018) posit that mind wandering is better understood as a series of distinct phenomena united by family resemblances, rather than a uniform mechanism, and thus neural indicators may differ depending on the context or experience sampling method used. Our findings thus suggest that these oscillatory markers are reliable differentiators of mind wandering that are applicable in applied settings that do not require a button press method and require participants to remain vigilant for extended periods of time.

A limitation to our findings was that task load was not significantly associated with either mind wandering or learning. The cognitive theory of multimedia learning (Mayer and Moreno, 2003) posits that task load generated by extraneous factors inhibits learning but that a moderate degree of cognitive load facilitates learning. On the other hand, it is possible that the present task was not sufficiently demanding that this influenced learning; some findings suggest that there is a u-shaped relationship between mind wandering and mental workload (Sullivan and Davis, 2020), though this requires further research. Regardless of these limitations, the findings overall suggest that attention is redirected away from videos and toward external stimuli during periods of mind wandering during online lecture use, and that this may explain the negative impact of mind wandering in learning environments. E-learning technology users may benefit from techniques which limit mind wandering. Developers of such technologies may wish to consider factors which limit mind wandering in multimedia and curriculum design, such as with active learning techniques, or by employing a blend of both synchronous and asynchronous content.

## Data Availability Statement

The datasets presented in this article are not readily available because consent from participants was not given to share this data publicly. Scripts and output used in this analysis are publicly available at https://github.com/cdconrad/aalis. Requests to access the datasets should be directed to the corresponding author, colin.conrad@dal.ca.

## Ethics Statement

The studies involving human participants were reviewed and approved by the Dalhousie University Research Ethics Board. The patients/participants provided their written informed consent to participate in this study.

## Author Contributions

CC designed the study and analyzed the data under the supervision of AN. CC collected the data. CC and AN wrote the final manuscript. Both authors contributed to the article and approved the submitted version.

## Conflict of Interest

The authors declare that the research was conducted in the absence of any commercial or financial relationships that could be construed as a potential conflict of interest.

## Publisher’s Note

All claims expressed in this article are solely those of the authors and do not necessarily represent those of their affiliated organizations, or those of the publisher, the editors and the reviewers. Any product that may be evaluated in this article, or claim that may be made by its manufacturer, is not guaranteed or endorsed by the publisher.
